# GRIA1 regulates TGN export and secretion of Sonic hedgehog

**DOI:** 10.1016/j.jbc.2025.111084

**Published:** 2025-12-22

**Authors:** Xiao Tang, Ye Tian, Qianyuan Wang, Ziyang Song, Xiaoxu Zhao, Yusong Guo

**Affiliations:** 1 Anhui Provincial Key Laboratory of Molecular Enzymology and Mechanism of Major Metabolic Diseases, College of Life Sciences, Anhui Normal University, Wuhu, China; 2 Division of Life Science, The Hong Kong University of Science and Technology, Hong Kong, China

**Keywords:** Sonic hedgehog, GRIA1, protein secretion, TGN export, clathrin

## Abstract

Sonic hedgehog (Shh) signaling orchestrates diverse developmental processes in metazoans and is implicated in numerous human diseases. While downstream signaling in recipient cells have been extensively characterized, the mechanisms governing secretion of newly synthesized Shh from producer cells remain less well understood. Building on our previous identification of a Surfeit locus protein 4 (SURF4)-to-proteoglycan (PG) relay mechanism that mediates endoplasmic reticulum (ER)–to–Golgi transport of the N-terminal Shh fragment (ShhN), we investigated ShhN export from the trans-Golgi network (TGN). We show that ShhN exits the TGN *via* a clathrin-dependent secretory pathway. Mechanistic analyses identify the transmembrane protein glutamate receptor 1 (GRIA1) as a key mediator: GRIA1 associates with ShhN in Golgi-derived vesicles, physically interacts with ShhN, colocalizes with ShhN after TGN exit, and is required for efficient TGN export and secretion of ShhN. Notably, the Cardin–Weintraub (CW) motif on ShhN, previously shown to engage SURF4 for ER–to–Golgi trafficking, is also essential for TGN export, and PGs are critical for the GRIA1–ShhN interaction. Furthermore, GRIA1 regulates intracellular trafficking of endogenous full length Shh and modulates Shh pathway activity in Neuro-2a (N2A) cells. Together, these findings identify GRIA1 as an important regulator of Shh TGN export and advance our understanding of the molecular mechanisms that control Shh secretion.

Sonic hedgehog (Shh), a member of Hedgehog (Hh) family, is an important signaling molecule that regulates various developmental processes in metazoans, including cell proliferation, cell differentiation, embryonic patterning and organogenesis (1, 2, 3). Shh signaling pathway also contributes to the growth and migration of cancer cells. Aberrant Shh signaling has been implicated in the development of numerous human diseases and cancers (4).

Shh is synthesized as a full-length precursor protein (Shh^FL^) that enters the endoplasmic reticulum (ER) under the guidance of N-terminal signal peptide. Following cleavage of the signal peptide, the ∼45 kDa Shh^FL^ undergoes autocatalytic processing to generate two fragments: the N-terminal truncated form (ShhN) (∼19 kDa) and the C-terminal truncated form (ShhC) (∼26 kDa) (5). Subsequently, ShhC is degraded through endoplasmic reticulum-associated degradation (ERAD) (6), while ShhN is recognized by the cargo receptor Surfeit locus protein 4 (SURF4) through its polybasic Cardin–Weintraub (CW) motif, leading to its packaging into COPII vesicles and subsequent delivery to the Golgi (7). After reaching the trans-Golgi network (TGN), ShhN is exported to the plasma membrane and secreted into the extracellular space. Ultimately, ShhN in the extracellular space is recognized by its receptor on the surface of target cells, initiating a signaling cascade that regulates the expression of target genes *via* the Gli family of transcription factors (8).

While the downstream signaling cascade activated by secreted Shh in target cells has been extensively investigated, relatively little is known about the mechanisms underlying the secretion of newly synthesized Shh from producing cells. Our previous research reported that SURF4 and proteoglycans (PGs) utilized a relay mechanism to deliver ShhN from the ER to the Golgi (7). However, the molecular mechanism regulating TGN export and post-Golgi trafficking of ShhN remains poorly understood. At the TGN, various transmembrane cargo receptors have been shown to mediate the enrichment of soluble cargo proteins into TGN-derived vesicles (9). For example, mannose-6-phosphate receptors (MPRs) recognize mannose-6-phosphate (M6P) modifications on lysosomal enzymes to transport them from the TGN to endosomes (10). Lysosomal integral membrane protein type 2 (LIMP-2) mediates the trafficking of β-glucocerebrosidase (βGC) *via* an MPR-independent pathway (11). Sortilin regulates the trafficking of lysosomal enzymes as well as non-lysosomal cargo proteins, including brain-derived neurotrophic factor, TRK family proteins and insulin-like growth factor 2 (IGF2) (12, 13, 14). Sortilin-related receptor with A-type repeats (SorLA) regulates the trafficking of amyloid precursor protein (APP), linking to Alzheimer’s disease (15). However, the identity of the cargo receptor responsible for sorting Shh at the TGN remained elusive.

Here, we analyzed the kinetics of post-Golgi trafficking of ShhN utilizing the Retention Using Selective Hook (RUSH) assay (16) and an *in vitro* vesicle reconstitution assay (17). Our findings indicate that ShhN is exported from the TGN *via* a clathrin-dependent secretory pathway. We identified that glutamate receptor 1 (GRIA1) associates with ShhN in Golgi-derived vesicles, interacts with ShhN and colocalizes with SBP-EGFP-ShhN following ShhN exit from the TGN. Maturation of PGs is essential for the interaction between ShhN and GRIA1. Deficiency of GRIA1 impairs the TGN export and secretion of ShhN, as well as two other polybasic motif-containing cargoes, bone morphogenetic protein 8A (BMP8A) and secreted frizzled-related protein 1 (SFRP1). Our study demonstrates that GRIA1 is a key regulator of Shh TGN export and consequently mediates Shh signaling pathway, providing insights into the molecular mechanisms governing the TGN export of ShhN.

## Results

### GRIA1 regulates TGN export of ShhN

First, to analyze TGN export of Shh, we performed the RUSH transport assay using ShhN as described previously (7, 18). The RUSH plasmid of ShhN encodes the following components: ShhN without the signal peptide (amino acid: 25–198) fused downstream of an N-terminal signal peptide derived from IL-2, the streptavidin-binding peptide (SBP) and enhanced green fluorescent protein (EGFP; SBP-EGFP-ShhN); and an ER retention signal (Lys-Asp-Glu-Leu; KDEL) fused downstream of streptavidin (Str-KDEL) (SI Appendix, Fig. S1*A*). HeLa cells were transfected with plasmids encoding Str-KDEL_SBP-EGFP-ShhN. SBP-EGFP-ShhN fusion protein was retained in the ER because of the interaction between SBP and streptavidin. 24 h after transfection, the cells were treated with biotin and incubated at 20 °C for 2 h. SBP-EGFP-ShhN were released from the ER and accumulated in the TGN (SI Appendix, Fig. S1, *A* and *C*). Then the cells were transferred to 37 °C for 45 min to release SBP-EGFP-ShhN from the TGN (SI Appendix, Fig. S1, *A* and *D*, magnified views in Fig. S1*D′*). Clathrin-coated vesicles (CCVs) play a crucial role in transporting proteins across various compartments within both the secretory and endocytic systems (19). To determine whether clathrin is important for the exit of Shh out of the TGN, we used small interfering RNA (siRNA) against clathrin heavy chain (CHC) to reduce the expression of CHC in HeLa cells (SI Appendix, Fig. S1*B*). We found that the number of punctate structures of SBP-EGFP-ShhN per cell was significantly reduced in CHC knockdown cells comparing with MOCK cells (SI Appendix, Fig. S1, *C*–*F* and quantification in Fig. S1*G*), indicating that TGN export of ShhN is clathrin-dependent.

To exclude the possibility that this effect resulted from a general disruption of TGN function, we examined whether CHC knockdown affects the export of TGN46, a well-established cargo of the Carriers of the TGN to the cell surface (CARTS) pathway (20, 21). Our results show that while CHC knockdown effectively blocks ShhN export from the Golgi, it did not affect the export of TGN46 (SI Appendix, Fig. S2, *A*–*C*), indicating that TGN secretory capacity is not broadly impaired by CHC knockdown. Critically, immunofluorescence analysis demonstrated a partial but significant colocalization between ShhN and CHC in post-TGN puncta after release at 37 °C (SI Appendix, Fig. S1, *H*–*J*). This colocalization, though transient due to the rapid uncoating of CCVs, provides direct visual evidence for ShhN being packaged into clathrin-coated carriers. To further test whether ShhN itself might be exported *via* CARTS, we performed immunofluorescence analysis and observed that ShhN and the CARTS marker TGN46 localize to distinct, largely non-overlapping puncta following release from the TGN (SI Appendix, Fig. S2, *D*–*K*), demonstrating that they are sorted into separate export carriers. Taken together, these findings provide strong evidence that ShhN is exported from the TGN *via* clathrin-coated vesicles, independently of the CARTS pathway.

We previously conducted co-immunoprecipitation (co-IP) experiments and label-free quantitative mass spectrometry analysis to identify proteins that interact with the HA-tagged ShhN (ShhN-HA) (7). A transmembrane protein, glutamate receptor 1 (GRIA1) was identified as a binding partner of ShhN rather than the control cargo protein, insulin growth factor like-2 (IGF2) (7). GRIA1 is a subunit of α-amino-3-hydroxy-5-methyl-4-isoxazole propionate receptors (AMPARs), which serve as excitatory receptors for the neurotransmitter L-glutamate in the central nervous system (CNS) (22). The glutamate receptor family in humans consists of four members, GRIA1, GRIA2, GRIA3 and GRIA4. GRIA1 subunits can form homomeric AMPARs or assemble into heteromeric AMPARs by combining with other subunits (23). AMPARs containing GRIA1 play crucial roles in brain function, particularly in synaptic transmission and plasticity, and have been implicated in neurological disorders, including schizophrenia (24). Despite its well-established role in excitatory neurotransmission, the involvement of GRIA1 in intracellular trafficking remains unexplored.

**Figure 1:**
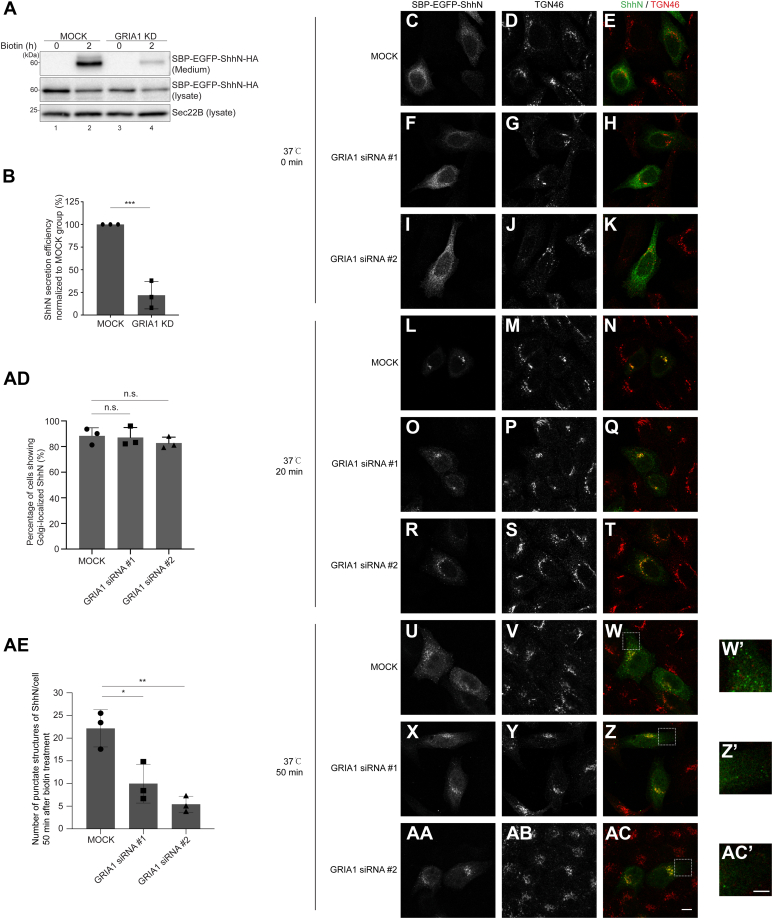
**GRIA1 regulates TGN export and the secretion of ShhN.***A*, HEK293T cells were transfected with control siRNA or siRNA against GRIA1. 48 h after transfection, cells were further transfected with plasmids encoding Str-KDEL_SBP-EGFP-ShhN-HA. On Day 3, after knockdown, cells were incubated with biotin for 2 h. After biotin incubation, the level of SBP-EGFP-ShhN-HA in the medium and in cell lysates was analyzed by immunoblotting with anti-HA. *B*, quantification of the abundance of secreted SBP-EGFP-ShhN-HA 2 h after biotin treatment, normalized to the abundance detected in the cell lysate fraction under biotin-free conditions (mean ± SD; n = 3). ∗∗∗*p* < 0.001. *C*–*AC*, HeLa cells were transfected with NC siRNA (*C*–*E*, *L*–*N* and *U*–W) or two different siRNAs against GRIA1 (*F*–*K*, *O*–*T* and *X*–*AC*). 48 h after transfection, cells were transfected with plasmids encoding Str-KDEL_SBP-EGFP-ShhN. On day 3 after knockdown, cells were treated with biotin and incubated at 37 °C for 0 min (*C*–*K*), 20 min (*L*–*T*) or 50 min (*U*–*AC*), and the localization of SBP-EGFP-ShhN and TGN46 was analyzed (Scale bar, 10 μm). The magnified views of the indicated area in panels (W, Z and AC) are shown in panels (W*′, Z' and* AC*′*) (Scale bar, 5 μm). *AD*, quantifications of the percentage of cells showing juxta-nuclear–accumulated EGFP signal after incubation with biotin for 20 min (mean ± SD; n = 3; >100 cells counted for each time point). n.s., not significant. *AE*, quantification of the number of punctate structures containing SBP-EGFP-ShhN per cell 50 min after biotin treatment (n = 3, mean ± SD, over 20 cells were quantified in each experimental group). ∗*p* < 0.05, ∗∗*p* < 0.01.

To test whether GRIA1 is important for the intracellular transport and secretion of ShhN, we utilized siRNA against GRIA1 to reduce the expression of GRIA1 (SI Appendix, Fig. S3A-B) and analyzed the impact on the secretion of ShhN. We found that GRIA1 knockdown significantly reduced the secretion efficiency of the RUSH construct of ShhN (Fig. 1*A*, compare lanes 2 and 4, and quantification in Fig. 1*B*).

Next, we investigated which step in ShhN secretion is regulated by GRIA1. We first checked whether knocking down GRIA1 impacts ER export of ShhN. SBP-EGFP-ShhN was retained in the ER before biotin treatment (Fig. 1, *C*–*K*). 20 min after biotin addition, SBP-EGFP-ShhN was localized at the juxta-nuclear area in the majority of cells, colocalized with Golgi marker, TGN46 (Fig. 1, *L*–*N*). The percentages of cells displaying a juxta-nuclear pattern of ShhN in MOCK group and GRIA1 knockdown group showed no significant difference (Fig. 1, *L*–*T*, Quantification in Fig. 1*AD*), indicating that deficiency of GRIA1 does not affect the export of ShhN from the ER. We then investigated whether GRIA1 regulates TGN export of ShhN. To address this, we incubated Str-KDEL_SBP-EGFP-ShhN-expressing cells in the presence of biotin for 50 min. We found that the number of punctate structures of SBP-EGFP-ShhN per cell was significantly reduced in GRIA1 knockdown cells compared to control cells (Fig. 1, *U*–*AC*, and quantification in Fig. 1*AE*). We utilized the RUSH assay to examine the trafficking of another soluble cargo, insulin growth factor like-2 (IGF2). Our results showed that TGN export and secretion of SBP-EGFP-IGF2 was unaffected by GRIA1 knockdown (SI Appendix, Fig. S4, *A*–*G*). These analyses suggest that GRIA1 plays an important role in TGN export of ShhN and consequently regulates ShhN secretion.

### GRIA1 mediates the packaging of ShhN into TGN-derived transport vesicles

**Figure 2:**
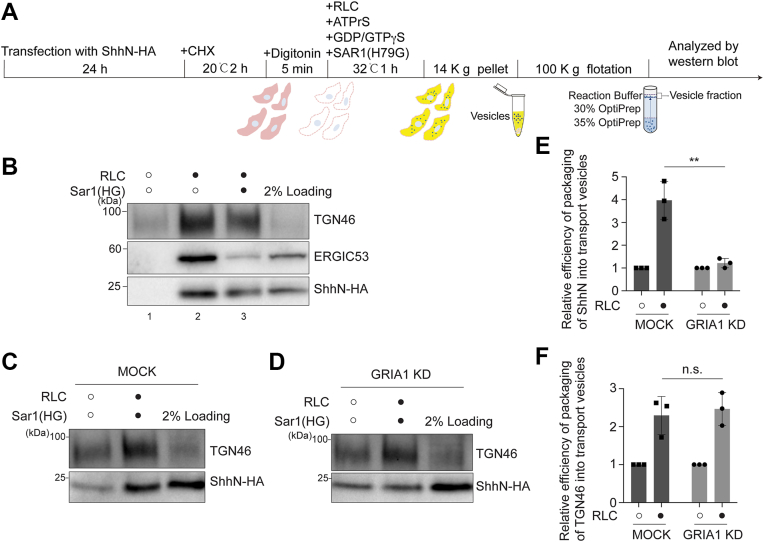
**GRIA1 regulates the packaging of ShhN into TGN-derived vesicles.***A*, diagram depicting the vesicle formation assay to reconstitute release of ShhN-HA into transport vesicles. *B*, HEK293T cells were transfected with plasmids encoding ShhN-HA. 24 h after transfection, vesicle formation was performed using the indicated reagents. The vesicle fraction was analyzed by immunoblotting using anti-TGN46, anti-ERGIC53 or anti-HA antibodies. *C* and *D*, HEK293T cells were transfected with NC siRNA (*C*) or siRNA against GRIA1 (*D*). At 48 h after transfection, cells were transfected with plasmids encoding ShhN-HA. On Day 3 after knockdown, vesicle formation was performed using the indicated reagents in MOCK cells (*C*) and GRIA1 KD cells (*D*). The vesicle fraction was analyzed by immunoblotting using anti-TGN46 or anti-HA antibodies. *E* and *F*, quantification of the percentage of ShhN-HA (*E*) or TGN46 (*F*) that was packaged into transport vesicles (n = 3, mean ± SD). ∗∗*p* < 0.01; n.s., not significant.

To investigate whether GRIA1 regulates the packaging of ShhN into TGN-derived vesicles, we reconstituted the vesicle formation from the TGN *in vitro* (25, 26). HEK 293T cells expressing ShhN-HA were incubated at 20 °C for 2 h in the presence of the protein synthesis inhibitor cycloheximide (CHX) to enrich ShhN-HA proteins at the TGN. Then we performed *in vitro* vesicular formation assay as previously described (7). Briefly, the cells were permeabilized by digitonin for 5 min. After that, the semi-intact cells were incubated at 32 °C for 1 h with rat liver cytosol (RLC), ATP regeneration system (ATPrS) and GTP. A GTP hydrolysis defective form of Sar1A, Sar1A (H79G), was also added into the reaction mix to inhibit the COPII vesicle formation. The vesicles released were collected by floatation and the proteins enriched in the vesicles were analyzed by immunoblotting (Fig. 2*A*). We found that ShhN-HA is enriched in transport vesicles in a cytosol-dependent manner (Fig. 2*B*, compare lanes 1 and 2). The addition of Sar1A (H79G) strongly impaired packaging of the canonical COPII client, ERGIC53, while ShhN-HA packaging was only mildly affected (Fig. 2*B*, compare lanes 2 and 3). We propose that most ShhN in these vesicles under Sar1A (H79G) treatment originates from TGN-derived vesicles. GRIA1 knockdown significantly reduced ShhN packaging into transport vesicles (Fig. 2, *C* and *D* and quantifications in Fig. 2*E*), indicating that GRIA1 plays a key role in packaging ShhN into TGN-derived vesicles.

### GRIA1 co-localizes with ShhN upon TGN exit

**Figure 3:**
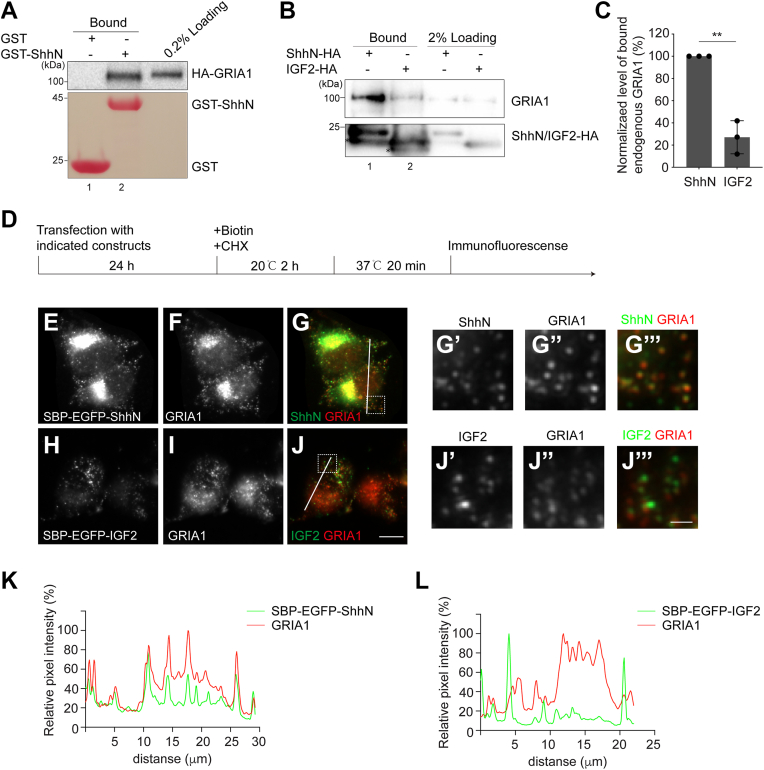
**GRIA1 interacts with ShhN and colocalizes with SBP-EGFP-ShhN following ShhN exit from the TGN.***A*, purified GST or GST-tagged ShhN was incubated with lysates from HEK293T cells transfected with HA-GRIA1. After incubation, the bound proteins were analyzed by immunoblotting with anti-HA antibodies. *B*, N2A cells were transfected with plasmids encoding the indicated constructs. Day 1 after transfection, cells were treated in 2 mM dithiobis (succinimidyl propionate) (DSP), and cell lysates were incubated with beads conjugated with HA antibodies. The bound proteins were analyzed by immunoblotting with anti-HA or anti-GRIA1 antibodies. ∗, non-specific bands. *C*, the percentage of GRIA1 that bound to IGF2-HA was normalized to that bound to ShhN-HA. The normalized abundance was then quantified (n = 3, mean ± SD). ∗∗*p* < 0.01. *D*, a diagram depicting the application of RUSH assay to analyze colocalization of cargo proteins and GRIA1. *E*–*J*, N2A cells were transfected with indicated plasmids. 1 day after transfection, cells were incubated at 20 °C for 2 h in the presence of biotin. The cells were then transferred to 32 °C for 20 min. After incubation, the localization of the indicated proteins was analyzed by immunofluorescence (Scale bar, 10 μm). Magnified views of the indicated area in panels G and J are shown in panels (G′–G′′′) and (*J′*–*J′′′*) (Scale bar, 2 μm). *K* and *L*, normalized intensity profiles are drawn from the white lines in (*G*) and (*J*) and show the relative pixel intensity along the line regarding the distance and fluorescence wavelength (*red*, GRIA1; *green*, ShhN-HA (*G*) or IGF2-HA (*J*)).

To test the interaction between GRIA1 and ShhN, we performed Glutathione S-transferase (GST) pull-down using purified GST-tagged ShhN (GST-ShhN) and HEK293T cells overexpressing HA-GRIA1. GST-ShhN interacted with HA-GRIA1, while GST alone did not (Fig. 3*A*, compare lanes 1 and 2). We also utilized the mouse neuroblastoma cell line Neuro-2a (N2A), which expresses high levels of endogenous Shh and GRIA1 (27, 28), to perform coimmunoprecipitation. We found that the abundance of endogenous GRIA1 that bound to ShhN-HA was significantly higher than that bound to IGF2-HA (Fig. 3*B*, compare lanes 1 and 2, and quantification in Fig. 3*C*), confirming the interaction between GRIA1 and ShhN.

Next, we examined the localization of GRIA1 and ShhN following ShhN exit from the TGN in N2A cells. Endogenous GRIA1 is localized in punctate structures and shows partial colocalization with the Golgi marker GM130 under steady-state conditions (SI Appendix, Fig. S7, *A*–*D*). N2A cells expressing Str-KDEL_SBP-EGFP-ShhN were treated with biotin and incubated at 20 °C for 2 h to release cargo proteins from the ER to the Golgi. The cells were then transferred to 37 °C to generate Golgi-derived vesicles. Following incubation, immunofluorescence was performed to analyze the localization of SBP-EGFP-ShhN and GRIA1 (Fig. 3*D*). After 37 °C incubation, we observed punctate localization of SBP-EGFP-ShhN and SBP-EGFP-IGF2 in the cell periphery (Fig. 3E and H). GRIA1 colocalized with SBP-EGFP-ShhN in Golgi-derived vesicles, but not with SBP-EGFP-IGF2 (Fig. 3, *E*–*L*, magnified views in Fig. 3, *G*–*G′′′* and *J′*–*J′′′*). These analyses indicate that SBP-EGFP-ShhN is packaged together with GRIA1 into transport vesicles at the Golgi, providing further insight into the role of GRIA1 in regulating TGN export of Shh.

### PG plays an essential role in mediating the association between Shh and GRIA1

**Figure 4:**
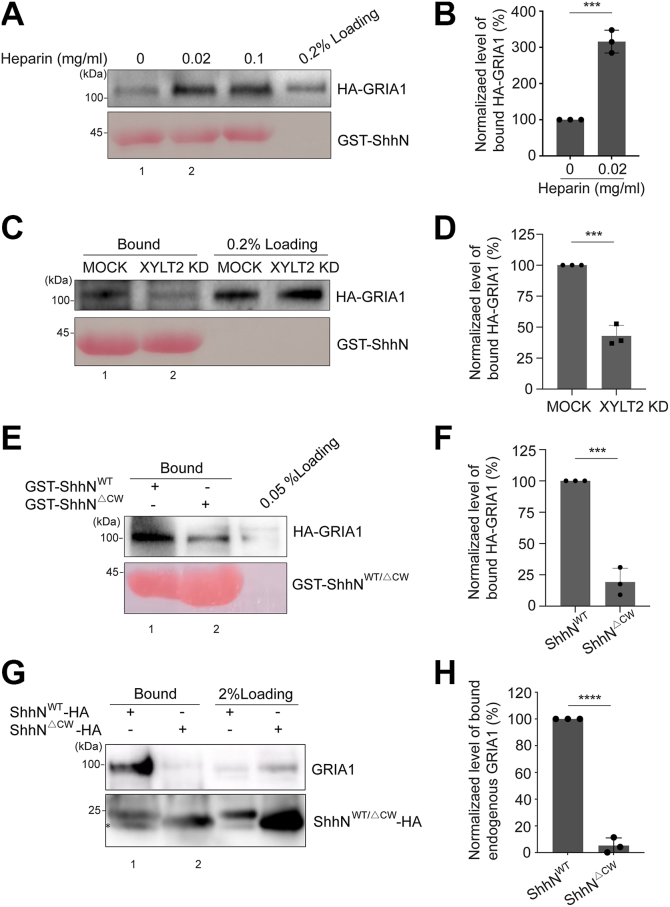
**GRIA1 interacts with ShhN in a PG-dependent manner.***A*, purified GST-ShhN was incubated with lysates from HEK293T cells transfected with HA-GRIA1 in the presence of the indicated concentrations of heparin. After incubation, the bound proteins were analyzed by immunoblotting with anti-HA antibodies. *B*, relative levels of HA-GRIA1 that bound to GST-ShhN were quantified (n = 3, mean ± SD). The quantification is normalized to the level of HA-GRIA1 that bound to GST-ShhN in the absence of heparin. ∗∗∗*p* < 0.001. *C*, HEK293T cells were transfected with negative control (NC) siRNA or siRNAs against XYLT2. At 48 h after transfection, cells were re-transfected with HA-GRIA1. On day 3 after knockdown, cell lysates were incubated with purified GST-ShhN. After incubation, the bound proteins were analyzed by immunoblotting with anti-HA antibodies. *D*, relative levels of HA-GRIA1 that bound to GST-ShhN were quantified (n = 3, mean ± SD). The quantification is normalized to the level of HA-GRIA1 that bound to GST-ShhN in MOCK group. ∗∗∗*p* < 0.001. *E*, purified GST-ShhN^WT^ or GST-ShhN^ΔCW^ were incubated with lysates from HEK293T cells transfected with HA-GRIA1. After incubation, the bound proteins were analyzed by immunoblotting with anti-HA antibodies. *F*, relative levels of HA-GRIA1 that bound to GST-ShhN^WT^ or GST-ShhN^ΔCW^ were quantified (n = 3, mean ± SD). The quantification is normalized to the level of HA-GRIA1 that bound to GST-ShhN^WT^. ∗∗∗*p* < 0.001. *G*, N2A cells were transfected with plasmids encoding the indicated constructs. 1 day after transfection, cells were treated in 2 mM dithiobis(succinimidyl propionate) (DSP), and cell lysates were incubated with beads conjugated with HA antibodies. The bound proteins were analyzed by immunoblotting with anti-HA or anti-GRIA1 antibodies. ∗, non-specific bands. *H*, the percentage of GRIA1 that bound to ShhN^ΔCW^-HA was normalized to that bound to ShhN^WT^-HA. The normalized abundance was then quantified (n = 3, mean ± SD). ∗∗∗∗*p* < 0.0001.

The core protein of proteoglycan (PG) is attached by glycosaminoglycan (GAG) chains at Golgi to form mature PG. We previously reported that PGs compete with SURF4, which is the cargo receptor of Shh at ER, to bind ShhN and the maturation of PGs is important for export of ShhN out of the TGN (7). These findings prompted us to hypothesize that PG may act as a scaffold to facilitate the formation of a Shh-PG-GRIA1 complex, thereby promoting TGN export of Shh. To test this, we first investigated whether PG and GRIA1 synergistically regulate TGN export of ShhN. We included a GAG, heparin, into GST pull down assay and found that the addition of heparin enhanced the interaction between ShhN and GRIA1 (Fig. 4*A*, compare lanes 1 and 2, and quantification in Fig. 4*B*). Consistent with a direct role for PG, we found that GRIA1 binds heparin in a manner dependent on its N-terminal heparan sulfate-binding motif (RRQR), as mutation of this motif, RRQR-to-AAQA, severely impaired the interaction (SI Appendix, Fig. S5*A*, compare lanes 1 and 2, and quantification in Fig. S5*B*). Meanwhile, we performed an siRNA knockdown experiment to reduce the expression of xylosyltransferase 2 (XYLT2), which is an enzyme catalyzes the attachment of GAG chains to PG core proteins, to inhibit the maturation of PGs (7). We found that in XYLT2 knockdown cells, the abundance of HA-GRIA1 that associated with GST-ShhN was reduced, comparing with MOCK cells (Fig. 4*C*, compare lanes 1 and 2, and quantification in Fig. 4*D*). These assays indicate that mature PG, *via* its GAG chains, interacts with GRIA1 through the RRQR motif and is essential for promoting the interaction between ShhN and GRIA1. This supports a model in which PG serves as a critical scaffold to nucleate a tertiary Shh-PG-GRIA1 export complex at the TGN.

We previously found that SURF4 and PG utilized a relay mechanism to deliver ShhN from the ER to the Golgi through interacting with polybasic CW motif (KRRHPKK) on ShhN (7). To test whether the CW motif is important for the interaction between GRIA1 and Shh, we performed GST-pull down assay using lysates from HEK293T cells expressing HA-GRIA1 and purified GST-ShhN^WT^. We found that GST-ShhN^WT^ interacted with HA-GRIA1 in cell lysates. Deleting the CW motifs in ShhN (GST-ShhN^ΔCW^) significantly reduced the binding between HA-GRIA1 and GST-ShhN (Fig. 4*E*, compare lanes 1 and 2, and quantification in Fig. 4*F*). To further validate these findings in endogenous system, we used N2A cells and observed similar results. The abundance of endogenous GRIA1 that bound to ShhN^ΔCW^-HA was significantly lower than that bound to ShhN^WT^-HA (Fig. 4*G*, compare lanes 1 and 2, and quantification in Fig. 4*H*). The interaction between certain soluble cargo proteins and their receptors can be influenced by varying pH levels in different intracellular compartments (29). The luminal pH of the ER is nearly neutral, while that of the TGN is approximately 6.0 (30). We found that reducing the pH from 7.2 to 6.0 did not cause a significant decrease in the interaction between ShhN and GRIA1 (data not shown). These analyses suggest that CW motif in Shh is critical for its interaction with GRIA1. Given that PG maturation is essential for the ShhN-GRIA1 interaction, and that Shh binds mature PG through its CW motif (31), we hypothesize that the PG-Shh interaction facilitates the binding of Shh to GRIA1, promoting the TGN export of Shh.

### The CW motif is sufficient for the GRIA1-mediated TGN export process

**Figure 5:**
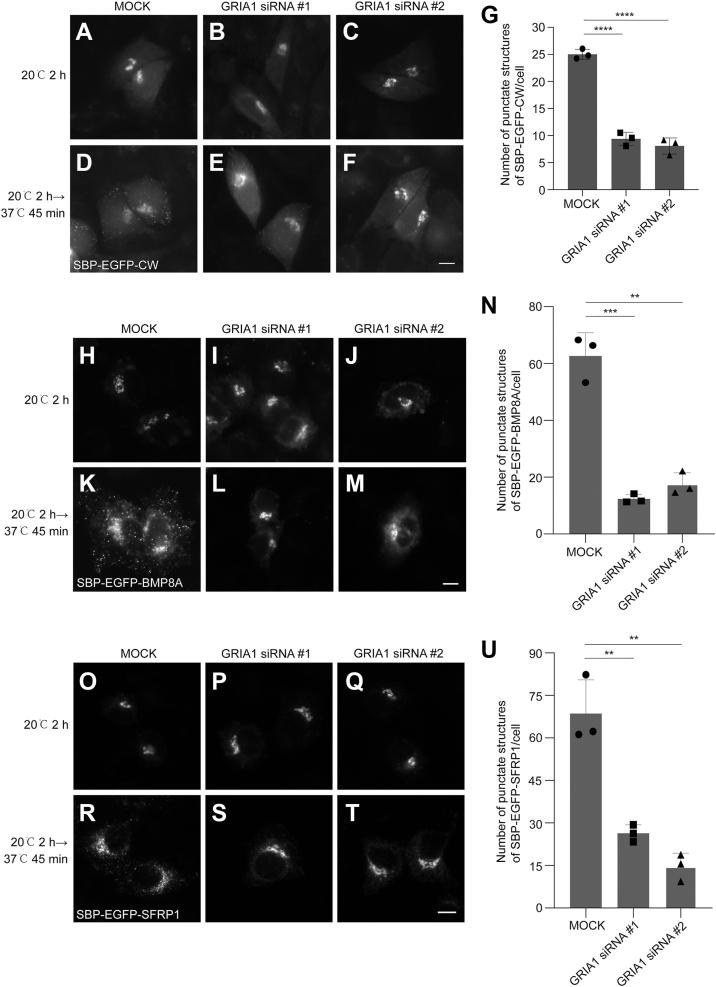
**The CW motif is sufficient for the GRIA1-mediated TGN export process.***A*–*F*, *H*–*M*, and *O*–*T*, HeLa cells were transfected with NC siRNA or two different siRNAs against GRIA1. 48 h after transfection, cells were transfected with plasmids encoding Str-KDEL_SBP-EGFP-CW (*A–F*), Str-KDEL_SBP-EGFP-BMP8A (*H*–*M*) or Str-KDEL_SBP-EGFP-SFRP1 (*O*–*T*). On Day 3 after knockdown, cells were treated with biotin and incubated at 20 °C for 2 h. Then, the cells were incubated at 37 °C for 0 or 45 min, and the localization of SBP-EGFP-CW was analyzed (Scale bar, 10 μm). (*G*, *N* and *U*) Quantifications of the number of punctate structures containing SBP-EGFP-CW (*G*), SBP-EGFP-BMP8A (*N*), or SBP-EGFP-SFRP1 (*U*) per cell 45 min after 37 °C incubation (n = 3, mean ± SD, over 20 cells were quantified in each experimental group). ∗∗*p* < 0.01; ∗∗∗*p* < 0.001; ∗∗∗∗*p* < 0.0001.

Disruption of the CW motif abolishes Shh-PG binding and consequently impairs the Shh-GRIA1 interaction, suggesting this motif also plays a critical role in TGN export of Shh. To investigate whether the CW motif is sufficient for the TGN export process, we fused CW motif downstream of SBP-EGFP in RUSH construct (Str-KDEL_SBP-EGFP-CW). RUSH construct containing no cargo protein sequence (Str-KDEL_SBP-EGFP) was retained in the ER after biotin treatment and temperature shift (SI Appendix, Fig. S6, *A* and *C*). In contrast, SBP-EGFP-CW was transported to the Golgi after 20 °C incubation and released from Golgi after 37 °C incubation (SI Appendix, Fig. S6, *B* and *D*, and quantification in Fig. S6, *E* and *F*). Notably, knockdown of GRIA1 also resulted in a delay in the export kinetics of SBP-EGFP-CW from the Golgi (Fig. 5, *A*–*F* and quantification in Fig. 5*G*). To further investigate whether GRIA1 is also crucial for the TGN export of other CW motif-like polybasic motif-bearing cargoes, we examined the impact of GRIA1 knockdown on TGN export of BMP8A and SFRP1, two cargoes contain polybasic motifs (18). We found that knockdown of GRIA1 significantly decreased the efficiency of TGN export of SBP-EGFP-BMP8A (Fig. 5, *H*–*M* and quantification in Fig. 5*N*) and SBP-EGFP-SFRP1 (Fig. 5, *O*–*T* and quantification in Fig. 5*U*). Importantly, both BMP8A and SFRP1 also directly bound to heparin, a proxy for PG GAG chains (SI Appendix, Fig. S5*C*). These findings indicate that GRIA1 plays a general role in regulating TGN export of cargo proteins containing polybasic motif, and the CW motif is sufficient for the GRIA1-mediated TGN export process.

### GRIA1 regulates the secretion of endogenous Shh and consequently mediates Shh signaling pathway

**Figure 6:**
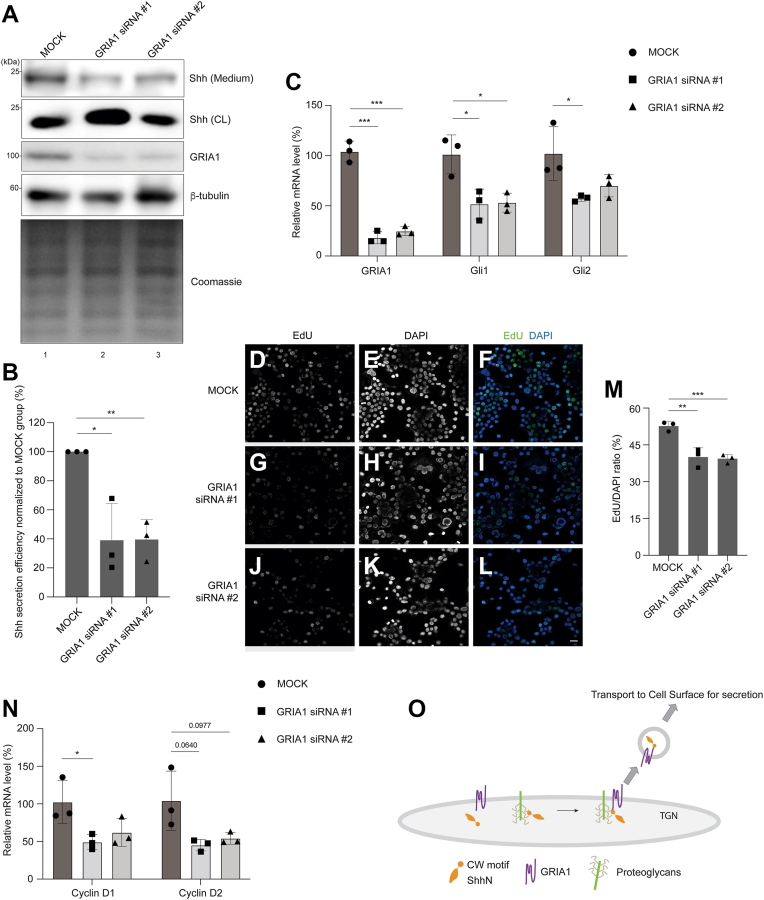
**GRIA1 regulates the secretion of endogenous Shh and consequently mediates Shh signaling pathway.***A*, N2A cells were transfected with NC siRNA or two different siRNAs against GRIA1. 72 h after transfection, the level of Shh in the medium and the levels of GRIA1, Shh and β-tubulin in cell lysates were analyzed by immunoblotting with anti-GRIA1, anti-Shh and anit-β-tubulin antibodies. The level of total protein in the medium was analyzed by Coomassie blue staining. *B*, quantification of the level of secreted Shh normalized to that detected in the MOCK group (mean ± SD; n = 3). In each experimental group, the secreted abundance of Shh is normalized to the abundance of Shh in cell lysates. ∗*p* < 0.05, ∗∗*p* < 0.01. *C*, N2A cells were transfected with NC siRNA or two different siRNAs against GRIA1. 72 h after transfection, the relative mRNA levels of GRIA1, Gli1 and Gli2 were analyzed by RT-qPCR (mean ± SD; n = 3). ∗*p* < 0.05, ∗∗∗*p* < 0.001. *D*–*L*, EdU proliferation assay was performed in N2A cells transfected with NC siRNA or two different siRNAs against GRIA1.The Edu signal and DAPI signal were analyzed by immunofluorescence (Scale bar, 10 μm). *M*, the number of cells showing EdU signal was quantified and normalized to the number of cells showing DAPI signal (n = 3, mean ± SD, over 800 cells were quantified in each experimental group). ∗∗*p* < 0.01; ∗∗∗*p* < 0.001. *N*, N2A cells were transfected with NC siRNA or two different siRNAs against GRIA1. 72 h after transfection, the relative mRNA levels of CyclinD1 and Cyclin D2 were analyzed by RT-qPCR (mean ± SD; n = 3). ∗*p* < 0.05. *O*, our proposed model depicting the molecular mechanisms that GRIA1 and PG regulate TGN export of Shh.

We then validated our findings in N2A cells, which naturally express high levels of endogenous Shh and GRIA1 (Fig. 6*A*). We utilized siRNA against mouse GRIA1 to reduce the protein level and mRNA level of GRIA1 in N2A cells (Fig. 6, *A* and *C*, and SI Appendix, Fig. S7, *A* and *E*). Knockdown of GRIA1 significantly decreased the level of endogenous Shh that secreted into medium (Fig. 6*A*, compare lanes 1–3, and quantification in Fig. 6*B*). To measure the effect of GRIA1 knockdown on Shh signaling pathway, we utilized quantitative real-time PCR (qPCR) to analyze the mRNA level of Gli1 and Gli2, which are the downstream components of Shh signaling pathway. We observed decreased mRNA levels of these genes (Fig. 6*C*), suggesting that GRIA1 knockdown downregulates Shh signaling pathway through suppressing the secretion of Shh.

Shh signaling pathway plays a crucial role in the growth and migration of cancer cells. Shh secreted by cancer cells is recognized by the receptors on the surface of target cells, inducing the synthesis and secretion of other soluble growth factors (32). These factors promote the growth and migration of cancer cells, thereby creating a positive feedback loop (33). To examine the effect of GRIA1 knockdown on cell proliferation, we utilized Edu proliferation assay. MOCK or GRIA1 knockdown N2A cells were incubated with EdU for 4 h at 37 °C and the EdU/DAPI ratio was used to represent the proliferation rate. We found that the proliferation rate was significantly reduced in GRIA1 knockdown cells comparing with MOCK cells (Fig. 6, *D*–*L* and quantification in 6M). In addition, we also found that the mRNA levels of cell cycle regulators, cyclin D1 and cyclin D2, were decreased in GRIA1 knockdown cells (Fig. 6*N*). These findings indicate that GRIA1 regulates cell proliferation by modulating Shh secretion.

## Discussion

The molecular mechanisms regulating Shh secretion remain a critical gap in our understanding of Shh signaling. While previous studies have focused on the downstream signaling cascades activated by secreted Shh molecules, the processes governing Shh trafficking and secretion within producing cells are less understood. In this study, we analyzed the post-Golgi trafficking of Shh. We identified GRIA1 as a critical regulator of Shh export from the TGN and consequently mediates Shh signaling pathway. At the TGN, GRIA1 interacts with Shh to package Shh into clathrin-coated vesicles, which are then exported from the TGN. The maturation of PGs is essential for the interaction between Shh and GRIA1 (Fig. 4, *A–D*). We also found that knockdown of GRIA1 decreases TGN export of BMP8A and SFRP1, two other cargoes containing polybasic motifs (Fig. 5, *H*–*U*). It underscores the potential of GRIA1 as a general cargo sorting regulator. We previously reported that SURF4 regulate the ER export of polybasic motif-containing cargo proteins, including Shh, Indian hedgehog (Ihh), Desert hedgehog (Dhh), BMP8A and SFRP1 (18). These findings indicate that SURF4 and GRIA1 potentially collaborate in regulating secretion of a spectrum of cargoes.

GRIA1 is previously known as a subunit of AMPARs involved in synaptic plasticity and excitatory neurotransmission (22). The discovery of GRIA1 as a mediator of ShhN export from the TGN expands our understanding of the non-neuronal functions of GRIA1. Based on our results, GRIA1 is now shown to play a direct role in intracellular trafficking, suggesting that certain neurotransmitter receptor subunits may have dual roles. Future work would explore whether other AMPAR subunits, such as GRIA2-4, similarly influence protein trafficking, particularly for secretory cargoes with polybasic motifs. As a key mediator of glutamatergic neurotransmission in the CNS, GRIA1-deficient mice exhibit a range of neuropsychiatric disorders, including schizophrenia-like behaviors, attention-deficit hyperactivity disorder (ADHD), mood disorder, and bipolar disorder (34, 35, 36). Shh signaling is also essential for CNS development, synaptic plasticity, and neuronal homeostasis. Shh-deficient mice exhibit loss of the ventral neural tube (37). Both GRIA1 and Shh play essential roles in establishing and maintaining neurodevelopmental pathways. Their interaction, as revealed by this study, suggests that disruptions in GRIA1-mediated Shh secretion may have compounding effects on neurodevelopment. GRIA1 deficiency may impair AMPAR signaling and Shh secretion simultaneously, exacerbating deficits in excitatory neurotransmission and Shh-dependent neurogenesis. It may explain why GRIA1-deficient mice exhibit a complex behavioral phenotype that spans multiple neuropsychiatric domains. This hypothesis needs further investigation. An important future direction is to investigate whether supplement of Shh partially rescue the neurodevelopmental deficits due to deficiency of GRIA1.

Our analyses show that the addition of heparin enhances the interaction between Shh and GRIA1, and defects in PG synthesis reduce this interaction. The requirement for PG maturation in facilitating Shh binding to GRIA1 underscores the importance of molecular coordination during Shh trafficking. How might PGs enhance the GRIA1-Shh interaction? We hypothesize that PGs enhance this interaction by modulating the local concentration and orientation of Shh molecules. PGs tether Shh to the TGN membrane, preventing its diffusion and effectively increasing the likelihood of GRIA1 binding to Shh. Crystal structure analyses have shown that heparan sulfate proteoglycan (HSPG) binds with both fibroblast growth factor (FGF) and FGF receptor (FGFR) through its heparan sulfate (HS) chains. The formation of FGF-FGFR-heparin ternary complex promotes FGFR dimerization and activates downstream signaling pathway (38). Similarly, glypican-3 (GPC3), a member of PGs, binds to both Wnt ligand and its receptor, Frizzled, stimulating the endocytosis of Wnt-Frizzled complex and inducing Wnt signaling pathway (39). It would be interesting to investigate which specific PGs mediate the interaction between Shh and GRIA1 and regulate the TGN sorting of ShhN.

In this study, we identified GRIA1 as a key regulator of Shh secretion using both exogenous and endogenous expression systems in mammalian cell lines. A crucial next step will be to validate our findings in animal models. Aberrant Shh signaling is a hallmark of many cancers, where it promotes tumor growth, invasion, and metastasis (40). Targeting GRIA1 may represent a novel therapeutic strategy for modulating Shh secretion in cancers that depend on Shh signaling for progression. The development of small-molecule inhibitors that disrupt the interaction between GRIA1 and ShhN holds significant potential as a therapeutic approach.

## Materials and methods

### Constructs, reagents, cell culture, transfection, and immunofluorescence

HeLa cells, HEK293T cells and N2A cell lines were kindly provided by the University of California-Berkeley Cell Culture Facility and were confirmed by short tandem repeat profiling. All cell lines were tested negative for *Mycoplasma* contamination. HeLa cells, HEK293T cells and N2A cells were cultured in Dulbecco's Modified Eagle Medium (DMEM) containing 10% fetal bovine serum and 1% penicillin streptomycin mix (Invitrogen).

The cDNA encoding mouse ShhN, human GRIA1, human IGF2, human BMP8A and human SFRP1 were ordered from BGI (Beijing, China). The plasmids encoding C-terminal 3xHA-tagged ShhN, N-terminal 3xHA-tagged GRIA1, C-terminal 3xHA-tagged IGF2, Str-KDEL_SBP-EGFP-ShhN (aa: 25–198), Str-KDEL_SBP-EGFP-IGF2 (aa: 25–180), Str-KDEL_SBP-EGFP-BMP8A (aa:20–402), Str-KDEL_SBP-EGFP-SFRP1 (aa:23–314), Str-KDEL_SBP-EGFP-CW, and truncated versions of ShhN were generated by standard molecular cloning procedures. The N-terminus of SBP-EGFP tag is followed by a signal sequence derived from IL-2 (16).

siRNAs against GRIA1 and XYLT2 were purchased from Gene create (Wuhan, China). The target sequence of the two siRNAs against human GRIA1 is GGTGGACTGTGAATCAGAA and CAGGAAACGTGCAGTTTAA respectively. The target sequence of the siRNA against mouse GRIA1 is GTGCTTCATCACTCCAAGTTT and GGAGGCAGAGGATTGACATAT. The target sequence of the siRNA against XYLT2 is CTGGTAGTGTGGAGCTTCA. The commercial antibodies were rabbit anti-HA (Cell Signaling, catalogue number 3724), mouse anti-CHC (Abcam, catalogue number ab2731), mouse anti-AP1g1 (Sigma-Aldrich, catalogue number A4200), sheep anti-TGN46 (BIO-RAD, catalogue number AHP500G), anti-β-tubulin (Proteintech, catalogue number 80713-1-RR), mouse anti-Shh (Stanta Cruz, catalogue number sc-365112) and mouse anti-GRIA1 (Proteintech, catalogue number 67642-1-Ig). Rabbit anti-SEC22B antibodies and rabbit anti-ERGIC53 antibodies for the immunoblot analyses were kindly provided by Prof. Randy Schekman (University of California).

Transfection of siRNA or DNA constructs into HeLa cells, HEK293T cells or N2A cells and immunofluorescence were performed as described previously (7). Images were acquired with Eclipse Ti Motorized Inverted Fluorescence Microscope (Nikon) equipped with an Andor Zyla 4.2 sCMOS camera (Andor Technology) or Leica SP8 Confocal Laser Scanning Microscope (Leica).

### RUSH assay

RUSH assays were performed by treating HeLa cells or N2A cells transfected with plasmids encoding Str-KDEL and SBP-EGFP-ShhN, SBP-EGFP-IGF2, SBP-EGFP-CW, SBP-EGFP-BMP8A, or SBP-EGFP-SFRP1 in a complete medium containing 40 μM biotin (Sigma-Aldrich) and 100 ng/μl cycloheximide (Sigma-Aldrich) at the indicated temperature for the indicated time. Cells were then fixed by 4% paraformaldehyde and mounted on glass slides by ProLong Gold Antifade Mountant with DAPI (Invitrogen) for microscope analysis. All image analyses were performed using Fiji (ImageJ) software. The number of punctate structures was quantified using the “Analyze Particles” function in Fiji (ImageJ). Prior to analysis, a consistent background subtraction was applied to all images. A fixed noise tolerance (prominence) value was used to robustly distinguish specific signals from background, and maxima at the image edges were excluded.

To analyze the secretion of ShhN or IGF2, HEK293T cells transfected with plasmids encoding Str-KDEL_SBP-EGFP-ShhN or Str-KDEL_SBP-EGFP-IGF2 were treated by 100 ng/μl cycloheximide and 40 μM biotin in medium without FBS addition for the indicated time. N2A cells were incubated with medium without FBS addition for the indicated time. Then the secreted proteins were collected by trichloroacetic acid precipitation. The cells were collected and lysed by HKT buffer (100 mM KCl, 20 mM Hepes, pH 7.2, 0.5% Triton X-100). The precipitated proteins and cell lysates were analyzed by immunoblotting.

### Immunoprecipitation, protein purification, and binding assay

Immunoprecipitation of HA-tagged ShhN was performed by treating N2A cells incubating with 1 × PBS containing 2 mM dithiobis (DSP) and 2 mM CaCl2 at room temperature for 30 min, and then quenched with 25 mM Tris-HCl, pH 7.5. 200 μl of 0.5 mg/ml cell lysates were incubated with 10 μl of compact anti-HA agarose affinity beads with mixing at 4 °C overnight. After incubation, the beads were washed 4 times with 1 ml of HK buffer (100 mM KCl, 20 mM Hepes, pH 7.2), and the bound material was analyzed by immunoblotting.

Purification of GST-tagged ShhN or truncated ShhN and GST-tagged GRIA1 was performed as described previously (41). GST pull-down assays were carried out with 10 μl of compact glutathione beads bearing around 5 μg of GST-tagged ShhN or truncated ShhN. The beads were incubated with 200 μl of 0.5 mg/ml of cell lysates from HEK293T cells transfected with HA-GRIA1 or N2A cells in HKT buffer with mixing at 4 °C overnight. After incubation, the beads were washed three times with 500 μl of HKT buffer and twice with 500 μl of HK buffer, and the bound material was analyzed by immunoblotting.

Heparin-binding assay was performed using heparin sepharose beads (Abcam, cat. no. ab193268). HEK 293T cells were transfected with the indicated plasmids. Day 1 after transfection, cells were lysed and incubated with heparin sepharose beads with mixing at 4 °C overnight. After incubation, the beads were washed 4 times with 1 ml of HK buffer, and the bound material was analyzed by immunoblotting.

Protein band intensities were quantified using Fiji (ImageJ) software. Prior to quantification, a background subtraction was applied. For each band of interest, a rectangular selection of identical size was drawn around it. The signal intensity of the protein of interest was normalized to that of the corresponding loading control from the same lane. For each experimental condition, data from at least three independent biological replicates were collected and are presented as the mean ± SD.

### *In vitro* vesicle formation assay

*In vitro* vesicular release assays were performed as described previously (17, 42). Briefly, Day 1 after transfection with plasmids encoding HA-tagged ShhN, HEK293T cells grown in one 10- cm dish at around 90% confluence were incubated with 100 ng/μl cycloheximide at 20 °C for 2 h. Then the cells were permeabilized in 3 ml of ice-cold KOAc buffer containing 40 μg/ml digitonin on ice for 5 min, and the semi-intact cells were then sedimented by centrifugation at 300*g* for 3 min at 4 °C. The cell pellets were washed twice with 1 ml of KOAc buffer and resuspended in 100 μl of KOAc buffer. The budding assay was performed by incubating semi-intact cells (around 0.02 OD/reaction) with 2 mg/ml of rat liver cytosol in a 100 μl reaction mixture containing 200 μM GTP and an ATP regeneration system in the presence or absence of 0.5 μg of Sar1A (H79G). After incubation at 32 °C for 1 h, the reaction mixture was centrifuged at 14,000*g* to remove cell debris and large membranes. The medium-speed supernatant was then centrifuged at 100,000*g* to sediment small vesicles. The pellet fraction was then analyzed by immunoblotting. For density gradient flotation assays, the pellet fraction was resuspended in 100 μl of 35% OptiPrep and overlaid with 700 μl of 30% OptiPrep and 30 μl of KOAc buffer. The samples were centrifuged at 55,000 rpm in a TLS55 rotor in a Beckman ultracentrifuge for 2 h at 4 °C. After centrifugation, fractions were collected from the top to the bottom of the tube, and the top fraction was analyzed by SDS-PAGE and immunoblotting.

### Quantitative real-time PCR (qPCR)

Total RNA was extracted from N2A cells by PureLink RNA Mini Kit (Thermo Fisher Scientific, catalogue number 12183018A) and 1 μg Total RNA was reverse-transcripted into cDNA using Applied Biosystems High-Capacity cDNA Reverse Transcription Kit (Thermo Fisher Scientific, catalogue number 4374966). Quantitative PCR reaction was performed using LightCycler 480 SYBR Green I Master kit (Roche, catalogue number 04707516001). All the procedures were conducted according to the manufacturer’s guide. Genes are calculated using 2–ΔΔCt method and normalized to Gadph. Primers targeting mouse Shh (fwd: AATGCCTTGGCCATCTCTGT, rvs: GCTCGACCCTCATAGTGTAGAGAC), mouse Gria1 (fwd: AACTCAGTGAGCAAGGCGTC, rvs: AGAGCACTGGTCTTGTCCTTAC), mouse Gli1 (fwd: GGAAGTCCTATTCACGCCTTGA, rvs: CAACCTTCTTGCTCACACATGTAAG), mouse Gli2 (fwd: TACCTCAACCCTGTGGATGC, rvs: CTACCAGCGAGTTGGGAGAG), mouse Cyclin D1 (fwd: TCCTCTCCAAAATGCCAGAG, rvs: GCAGGAGAGGAAGTTGTTGG) and mouse Cyclin D2 (fwd: TCGATGATTGCAACTGGAAG, rvs: AGAGCTTCGATTTGCTCCTG) were used.

### EdU proliferation assay

The EdU Cell Proliferation Kit (Thermo Fisher Scientific, catalogue number C10337) was used to visualize the newly replicated cells. N2A cells were incubated in 50 mM EdU for 4 h under growth conditions. After PBS wash, the cells were fixed with 4% paraformaldehyde for 15 min at room temperature (RT) and washed with PBS for five times. Then, the cells were incubated in permeabilization and blocking buffer (2.5% FBS, 0.1% Triton X100, 0.2 M Glycine in PBS) for 30 min at RT. Finally, the cells were incubated in EdU reaction solution (Thermo Fisher Scientific, catalogue number C10337) for 30 min at RT protected from light and washed with PBS for five times. DAPI was visualized using ProLong Gold Antifade Mountant (Invitrogen, catalogue number P36930).

## Data availability

All data needed to evaluate the conclusions in this paper are present within the article and its Supplementary Materials. Original datasets and analysis files are available from the corresponding author upon reasonable request.

## Supporting information

This article contains supporting information.

## Conflict of interest

The authors declare that they have no conflicts of interest with the contents of this article.
