# Supplementary material for: GRIA1 regulates TGN export and secretion of Sonic hedgehog
Source: J Biol Chem. 2025 Dec 22;302(2):111084. doi: 10.1016/j.jbc.2025.111084 (PMC12858347; doi:10.1016/j.jbc.2025.111084)
Supplement: Supporting Figures S1–S7 [file mmc1.docx]

**SI Appendix Figure S1-7**

**Figure S1**


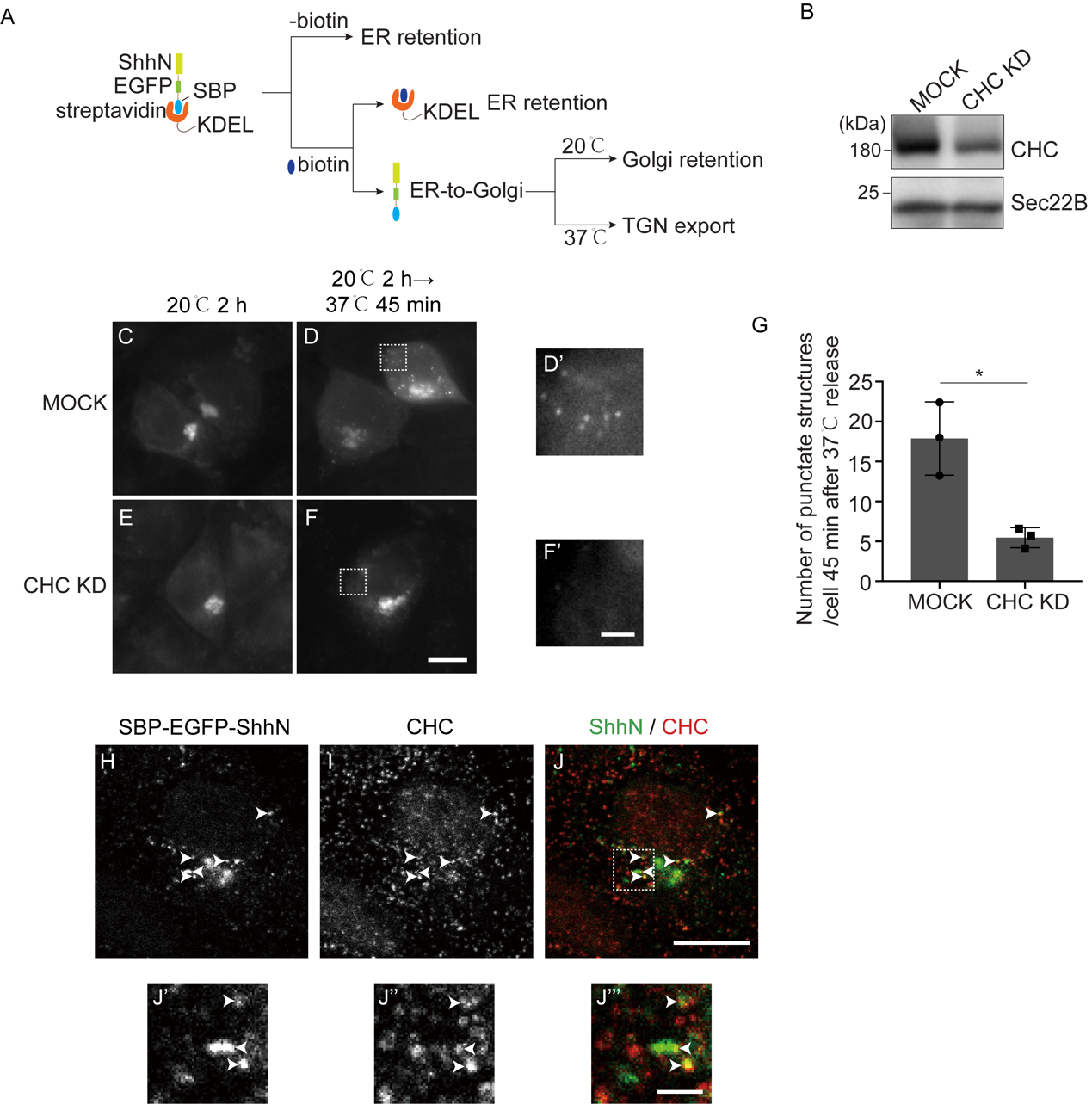


Figure S1. TGN export of ShhN depends on clathrin. (A) diagram depicting the application of RUSH assay to analyze secretory transport pathway of ShhN. (B) The level of CHC and SEC22B in cell lysates from HeLa cells transfected with NC siRNA or with siRNA against CHC were analyzed by immunoblotting with anti-CHC and anit-SEC22B antibodies. (C-F) HeLa cells were transfected with NC siRNA or siRNA against CHC. At 48 h after transfection, cells were transfected with plasmids encoding Str-KDEL and SBP-EGFP-ShhN. On day 3 after knockdown, cells were treated with biotin and incubated at 20 °C for 2 h. Then the cells were incubated at 37 °C for 0 or 45 min, and the localization of SBP-EGFP-ShhN was analyzed (Scale bar, 10 μm). The magnified views of the indicated area in panels D and F are shown in panels D’ and F’ (Scale bar, 2 μm). (G) Quantifications of the number of punctate structures containing SBP-EGFP-ShhN per cell 45 min after 37°C incubation (n = 3, mean ± SD, over 20 cells were quantified in each experimental group). *P < 0.05. (H-J) HeLa cells were transfected with Str-KDEL_SBP-EGFP-ShhN. 1 day after transfection, cells were incubated at 20 °C for 2 h in the presence of biotin. The cells were then transferred to 37 °C for 20 min. After incubation, the localization of the indicated proteins was analyzed by immunofluorescence (Scale bar, 10 μm). Magnified views of the indicated area in panel C are shown in panels C’-C’’’ (Scale bar, 2 μm).

**Figure S2**


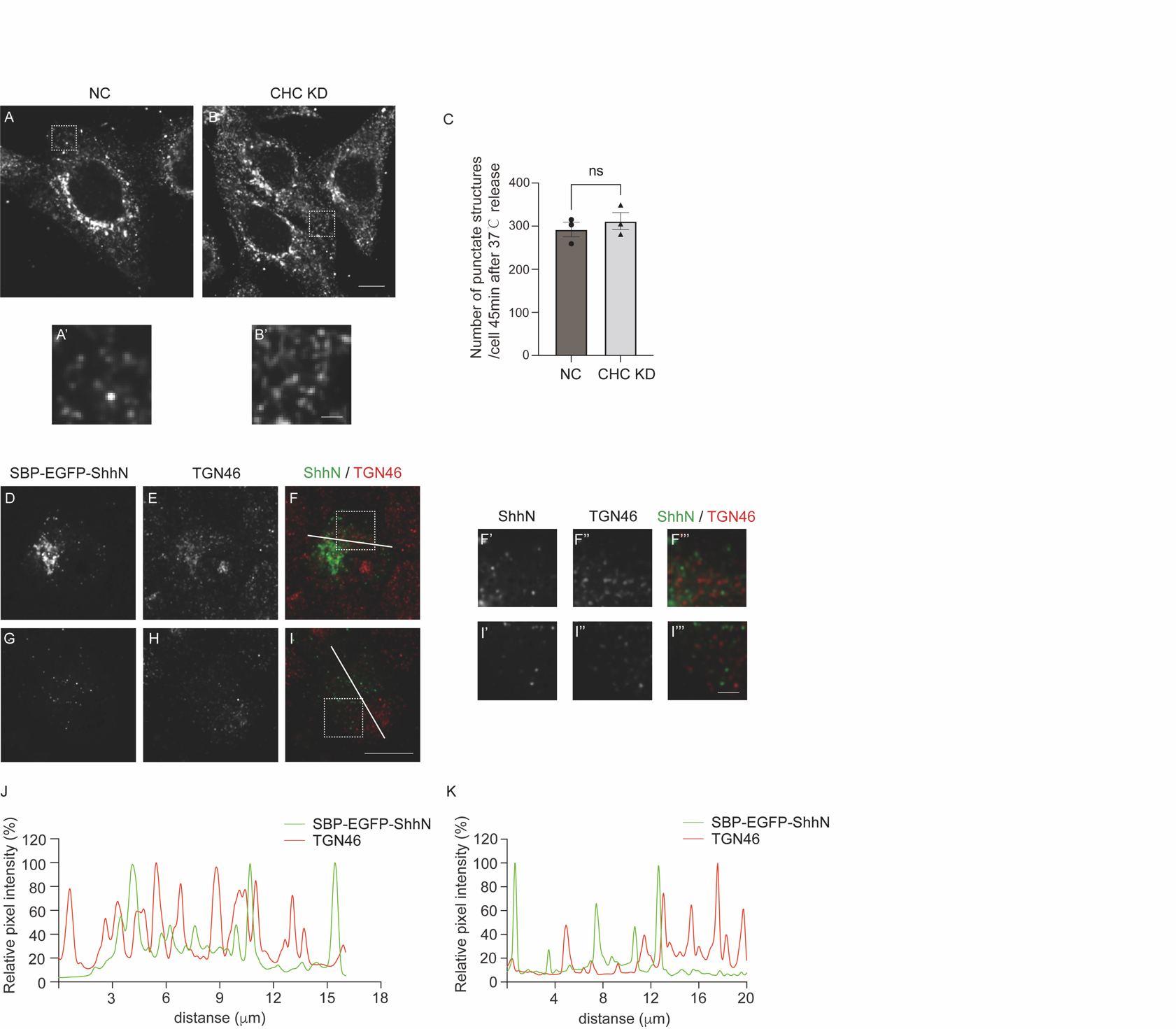


**Fig. S2. CHC knockdown has no significant effect on the Golgi export of CARTS client, TGN46.** (A-B) HeLa cells were transfected with NC siRNA (A) or siRNA against CHC (B). 72 h after knockdown, cells were treated incubated at 20 °C for 2 h. Then the cells were incubated at 37 °C for 45 min, and the localization of TGN46 was analyzed (Scale bar, 10 μm). The magnified views of the indicated area in panels A and B are shown in panels A’ and B’ (Scale bar, 2 μm). (C) Quantifications of the number of punctate structures containing TGN46 per cell 45 min after 37°C incubation (n = 3, mean ± SD, over 30 cells were quantified in each experimental group). ns, not significant. (D-I) N2A cells were transfected with Str-KDEL_SBP-EGFP-ShhN. 1 day after transfection, cells were incubated at 20 °C for 2 h in the presence of biotin. The cells were then transferred to 37 °C for 40 min. After incubation, the localization of the indicated proteins was analyzed by immunofluorescence (Scale bar, 10 μm). Magnified views of the indicated area in panels F and I are shown in panels F’-F’’’ and I’-I’’’ (Scale bar, 2 μm). (J-K) Normalized intensity profiles are drawn from the white lines in F and I and show the relative pixel intensity along the line regarding the distance and fluorescence wavelength (red, TGN46; green, SBP-EGFP-ShhN).

**Figure S3**


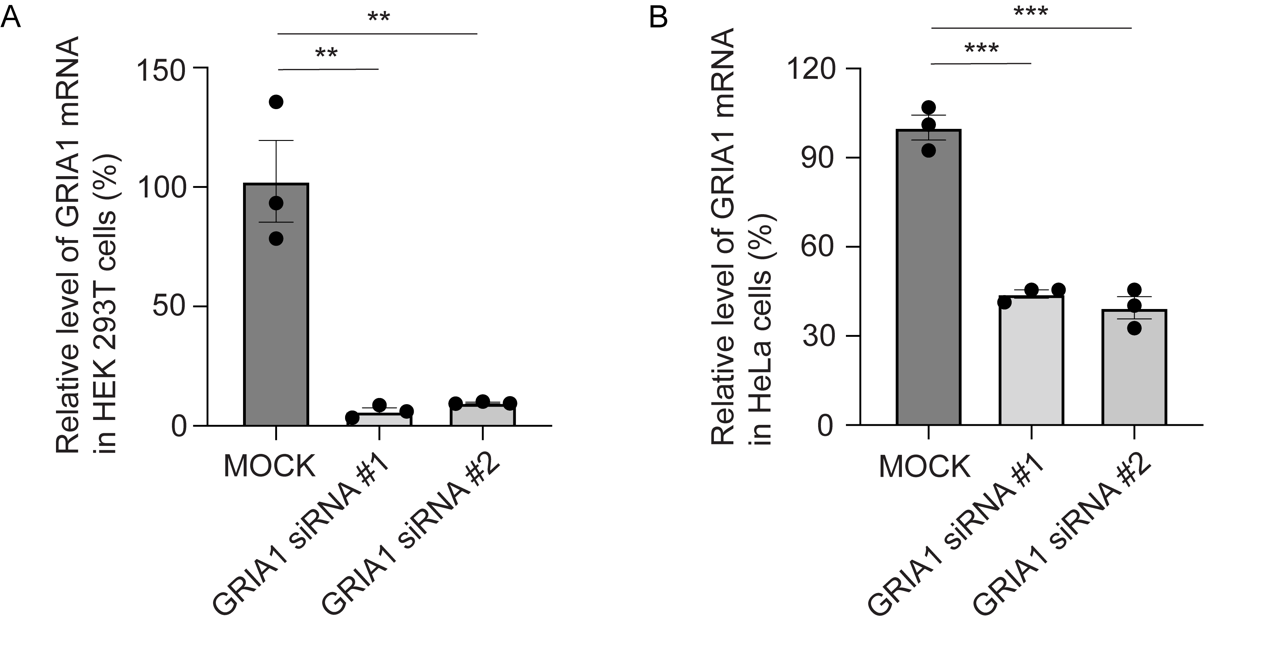


Figure S3. siRNAs against GRIA1 reduce mRNA level of GRIA1 in HEK293T cells and HeLa cells. (A-B) HEK293T cells (A) or HeLa cells (B) were transfected with negative control (NC) siRNA or two different siRNAs against GRIA1. 72 h after transfection, the relative mRNA levels of GRIA1 were analyzed by RT-qPCR (mean ± SD; n = 3). **P < 0.01, ***P < 0.001.

**Figure S4**


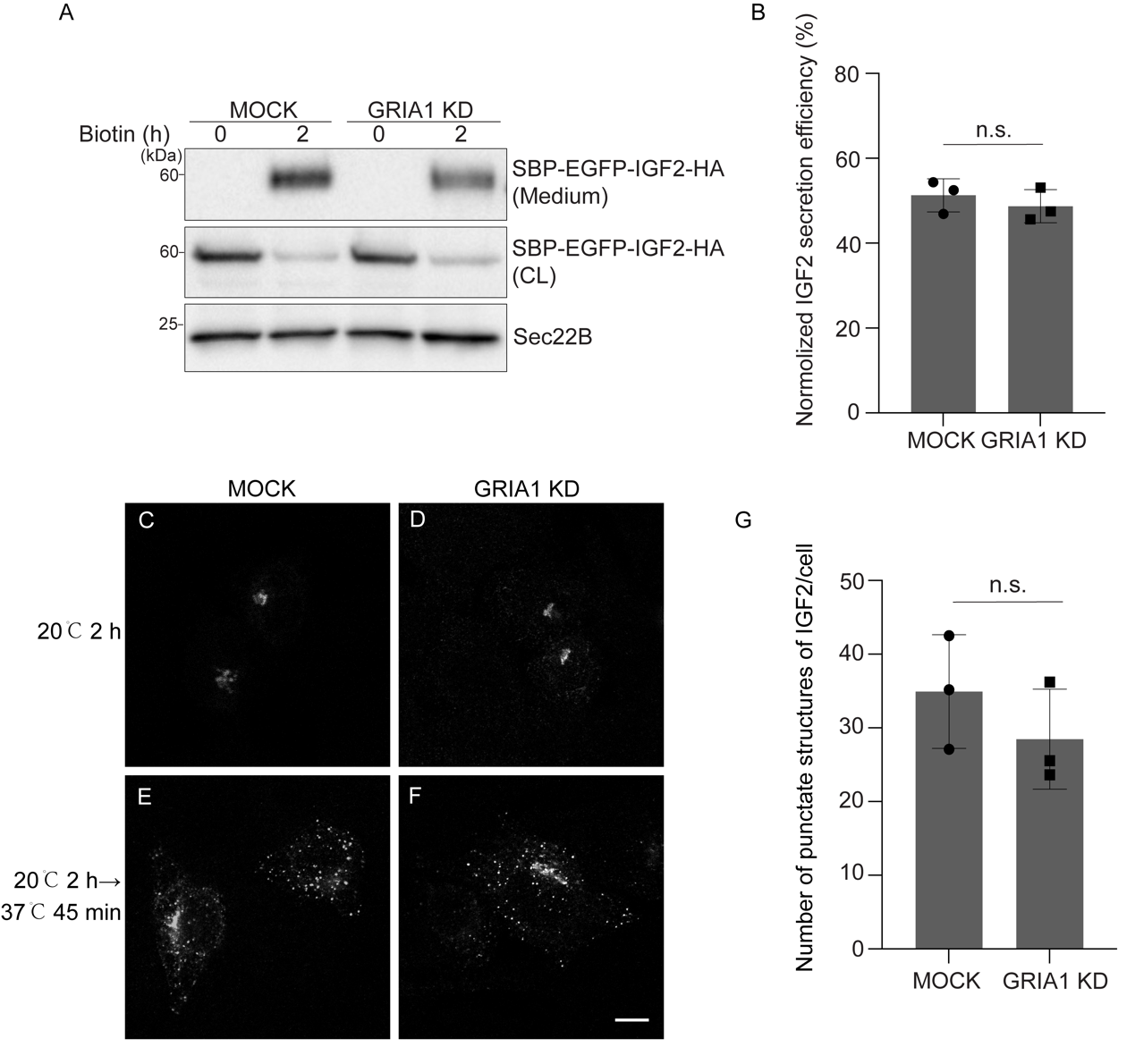


**Figure S4. Knockdown of GRIA1 does not cause defects in TGN export of IGF2.** (A) HEK293T cells were transfected with control siRNA or siRNA against GRIA1. 48 h after transfection, cells were re-transfected with plasmids encoding Str-KDEL and SBP-EGFP-IGF2-HA. On day 3 after knockdown, cells were incubated with biotin for 2 h, and the level of SBP-EGFP-IGF2-HA in the medium and in cell lysates were analyzed by immunoblotting using an anti-HA antibody. (B) Quantification of the abundance of secreted SBP-EGFP-IGF2-HA 2 h after biotin treatment normalized to the abundance detected in the cell lysate group in the absence of biotin (mean ± SD; n = 3). n.s., not significant. (C-F) HeLa cells were transfected with NC siRNA or siRNA against GRIA1. At 48 h after transfection, cells were transfected with plasmids encoding Str-KDEL and SBP-EGFP-IGF2. On day 3 after knockdown, cells were treated with biotin and incubated at 20 °C for 2 h. The cells were then shifted to 37 °C for 0 or 45 min, and the localization of SBP-EGFP-IGF2 was analyzed by fluorescence microscopy (Scale bar, 10 μm). (G) Quantifications of the number of punctate structures containing SBP-EGFP-IGF2 per cell 45 min after 37°C incubation (n = 3, mean ± SD, over 20 cells were quantified in each experimental group). n.s., not significant.

**Figure S5**

**
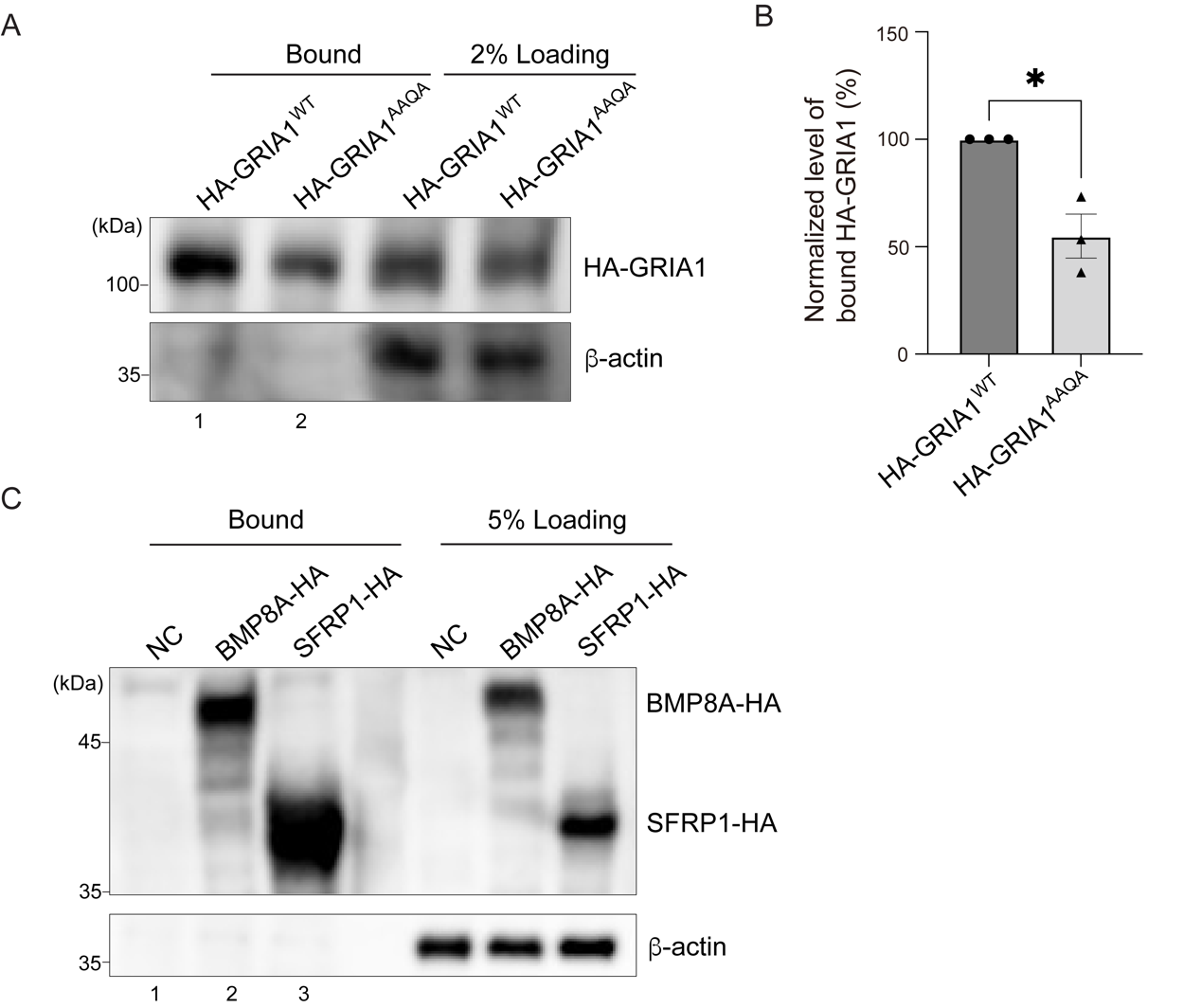
**

**Figure S5. Heparin binding assays assessing the binding of GRIA1, BMP8A and SFRP1 to heparin.** (A) Cell lysates from HEK 293T cells expressing HA-GRIA1^WT^ or HA-GRIA1^AAQA^ were incubated with heparin sepharose. After incubation, the bound proteins were analyzed by SDS-PAGE and immunoblotting. (B) Relative levels of HA-GRIA1^WT^ or HA-GRIA1^AAQA^ that bound to heparin were quantified (n = 3, mean ± SD). The quantification is normalized to the level of HA-GRIA1WT that bound to heparin. *P < 0.05. (C) Cell lysates from HEK 293T cells expressing BMP8A-HA or SFRP1-HA were incubated with heparin sepharose. After incubation, the bound proteins were analyzed by SDS-PAGE and immunoblotting.

**Figure S6**


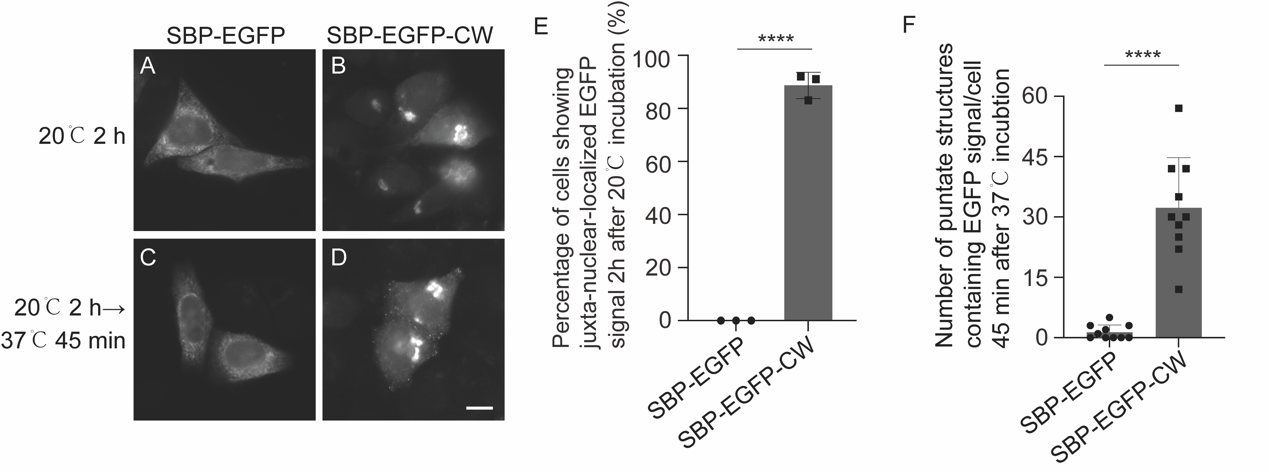


**Figure S6. Addition of CW motif to EGFP facilitates its ER and TGN export.** (A-D) HeLa cells were transfected with plasmids encoding Str-KDEL and SBP-EGFP-CW. On day 3 after knockdown, cells were treated with biotin and incubated at 20 °C for 2 h. Then the cells were incubated at 37 °C for 0 min (A-B) or 45 min (C-D), and the localization of SBP-EGFP-CW was analyzed (Scale bar, 10 μm). (E) Quantifications of the percentage of cells showing juxta-nuclear–accumulated EGFP signal after incubation with biotin for 2 h at 20 °C (mean ± SD; n = 3; >100 cells counted for each time point). n.s., not significant. ****P < 0.0001. (F) Quantification of the number of punctate structures containing EGFP signal per cell 45 min after 37°C incubation (mean ± SD, 10 cells were quantified in each experimental group). ****P < 0.0001.

**Figure S7**


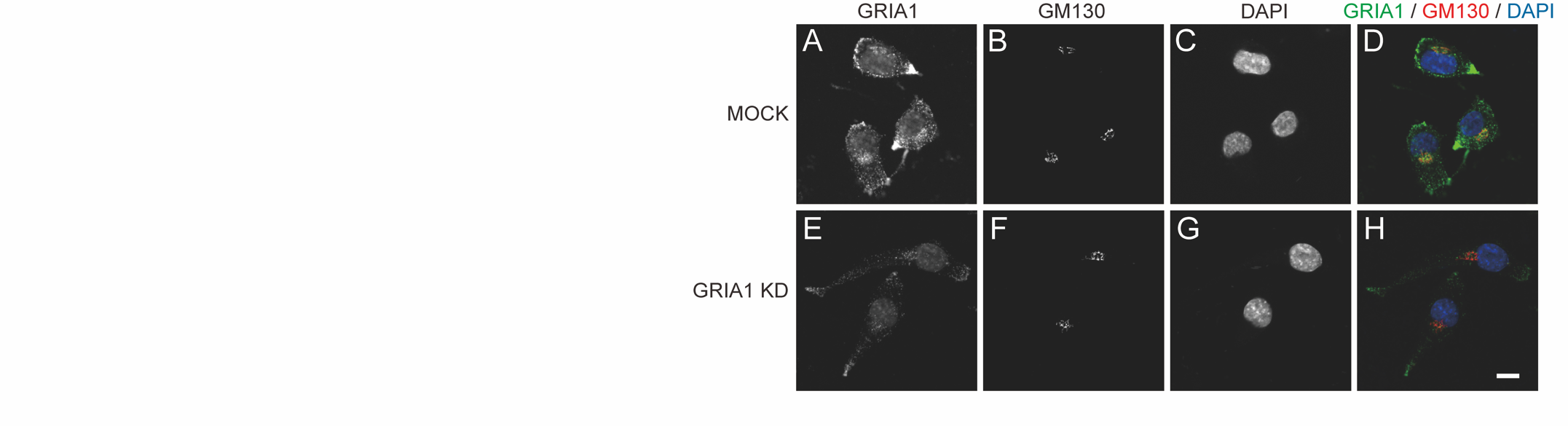


**Fig. S7. Localization of GRIA1 in MOCK and GRIA1 KD N2A cells.** (A-H) N2A cells were transfected with NC siRNA (A-D) or siRNA against GRIA1 (E-H). 72 h after transfection, the localization of GRIA1 was analyzed by fluorescence microscopy (Scale bar, 10 μm).
